# Accessibility explains preferred thiol-disulfide isomerization in a protein domain

**DOI:** 10.1038/s41598-017-07501-4

**Published:** 2017-08-29

**Authors:** Katra Kolšek, Camilo Aponte-Santamaría, Frauke Gräter

**Affiliations:** 1 Heidelberg Institute for Theoretical Studies, Heidelberg, Germany; 2 Interdisciplinary Center for Scientific Computing, Heidelberg University, Heidelberg, Germany; 3 Max Planck Tandem Group in Computational Biophysics, Present Address: University of Los Andes, Bogota, Colombia

## Abstract

Disulfide bonds are key stabilizing and yet potentially labile cross-links in proteins. While spontaneous disulfide rearrangement through thiol-disulfide exchange is increasingly recognized to play an important physiological role, its molecular determinants are still largely unknown. Here, we used a novel hybrid Monte Carlo and Molecular Dynamics scheme to elucidate the molecular principles of thiol-disulfide exchange in proteins, for a mutated immunoglobulin domain as a model system. Unexpectedly, using simple proximity as the criterion for thiol-disulfide exchange, our method correctly predicts the experimentally observed regiospecificity and selectivity of the cysteine-rich protein. While redox reactivity has been examined primarily on the level of transition states and activation barriers, our results argue for accessibility of the disulfide by the attacking thiol given the highly dynamic and sterically demanding protein as a major bottleneck of thiol-disulfide exchange. This scenario may be similarly at play in other proteins with or without an evolutionarily designed active site.

## Introduction

**Figure 1:**
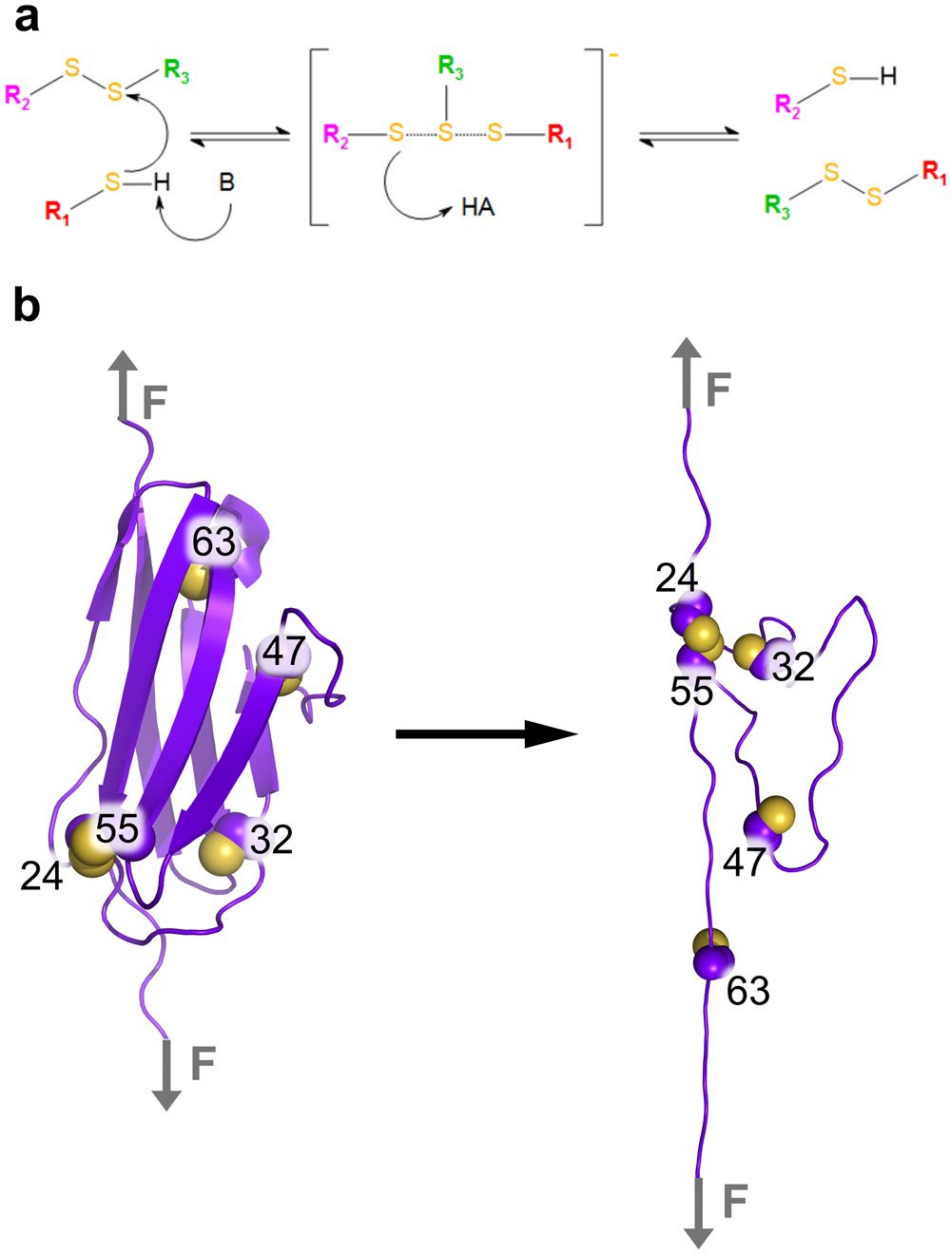
(**a**) Scheme of acid-base catalyzed thiol-disulfide exchange reaction proceeding through a classical S_N_2 trigonal bipyramidal transition state. (**b**) Schematic representation of force-clamp MD simulations during which the I27* domain unfolded under constant force application. The five cysteine residues contained in I27* are highlighted (carbon alpha and carbon beta atoms: purple spheres and sulfur atom: yellow sphere). The disulfide bond between 24Cys and 55Cys prevented the protein from fully stretching and stabilized a closed disorder loop. This loop confined two of the three free cysteines (32Cys and 47Cys) in proximity to the 24Cys–55Cys disulfide bond. The third free cysteine, 63Cys, at the C-terminal part of the protein moved away from the disulfide bond upon stretching.

Disulfide bonds are important post-translational modifications with many physiological roles. In proteins, disulfide bridges stabilize the tertiary and quaternary structure by lowering the configurational entropy of the unfolded form^1, 2^, and thereby crucially intervene in processes such as protein folding, assembly, catalysis, and protein regulation^3–6^. A disulfide bond is formed between two cysteines upon oxidation of the cysteine thiol groups. Contrary to other covalent bonds in proteins, disulfide bonds are reactive. They can undergo bimolecular nucleophilic substitution, S_N_2, a reaction with free thiol resulting in thiol-disulfide exchange. An activated thiol as a nucleophile attacks one of the two sulfurs of a disulfide bond to produce a new disulfide bond and a new free thiol (Fig. 1a). This reaction is of profound importance, as it is responsible for the enzymatic formation and opening of most protein disulfide bonds.

Recent evidence is mounting towards a highly dynamic nature of disulfide bonds in proteins. Disulfides do not only statically stabilize protein structure, but also rearrange due to spontaneous disulfide reshuffling. Disulfide reshuffling is common in the oxidative folding of proteins^7–9^. During protein folding disulfide crosslinks are formed *de novo* by external proteins or small molecules. This disulfide arrangement is not necessarily stable, and the folding intermediate undergoes disulfide bond reshuffling to reach the stable native fold with correct disulfide crosslinks^9–11^.

Intramolecular disulfide shuffling can also occur upon an external stimulus and thereby can change protein conformation and possibly function^12, 13^. For example, alpha-keratin, a structural protein abundant in hair and nails, reshuffles the interchain disulfide bonds upon mechanical stress (e.g. curling your hair), leading to protein conformational changes^14–16^. A similar example of intramolecular shuffling occurs in the von Willebrand factor protein. This enormous multimer is critically involved in primary hemostasis and is particularly rich in cysteines and disulfide bonds. Current data points toward disulfide shuffling triggered by shearing of the protein^14, 17, 18^.

Thus, disulfide bonds can readily react with thiols within proteins at physiologically relevant time scales even in the absence of enzymes. Given the multitude of possible thiol-disulfide exchange reactions, an important but hitherto unresolved question is how specificity is achieved. To uncover specific intramolecular thiol-disulfide exchange reactions at high resolution and at the single molecule level, Alegre-Cebollada *et al*.^19^ performed force-clamp atomic-force microscopy (AFM) on a mutated immunoglobulin I27 domain (I27*). They monitored the unfolding and putative thiol-disulfide exchange reactions in real time. These pioneering experiments allowed characterizing the kinetics of spontaneous intramolecular disulfide bond isomerization reactions that compete within the I27* protein^19^. Remarkably, the shuffling was highly specific, with only one of the free thiols attacking the bond, and this even with a clear preference for one of the two sulfurs within the bond. It remains unclear if spontaneous thiol-disulfide bond exchange is largely determined by the mere accessibility of the disulfide bridge for the thiol or if it is controlled by subtle details of the environment of the reaction center, such as the electrostatics.

Reactivity of thiol-disulfide exchange reactions has been extensively studied by computational methods in small molecules^20–23^ and in proteins, among others, thioredoxins^24^ and β3 integrin^25^. A major conclusion is that reactions are critically fine-tuned by the atomistic details of the active site. A prime example is the hydrophobic pocket around the CXXC motif of the thioredoxin family, the geometry, dynamics and electrostatic environment of which decide on the redox potential and kinetics^26, 27^. Therefore, the question arises, what are the driving forces for a spontaneous isomerization event within a protein, which lacks an evolutionarily designed active site for tailored catalysis.

Here, we investigated the molecular determinants of the spontaneous intramolecular thiol-disulfide exchange reactions in the mutated I27 domain as a model system. We used force-clamp molecular dynamics (MD) simulations (Fig. 1b) coupled with an energy based swapping criterion to simulate the dynamics of force-induced unfolding while allowing disulfide shuffling. Our simple, purely classical approach has unique advantages over the commonly used quantum mechanical methods to study thiol-disulfide exchange^20–22^. It does not require a priori definition of the reacting residues but instead can predict them, and it takes into account the large-scale protein motions and their coupling to the reaction at an atomistic level. By design it neglects the influence of the physical environment on the reaction barrier. Thus, it is reminiscent of MD simulations with reactive force fields such as ReaxFF^28^, which however do not reach the accuracy of current dedicated protein force fields.

Our results show the same regioselectivity and regiospecificity of the reactions observed by the experimental AFM setup^19^. Remarkably, a criterion based on the mere proximity of the attacking cysteine to the disulfide bond has the same predictive power as an energy-based Monte Carlo criterion. Our results suggest the accessibility of the bond-forming sulfur atoms to be sufficient to explain thiol-disulfide isomerization in this system, implying accessibility as a primary determinant of isomerization. Accessibility, in turn, is largely decided by the dynamic propensities of the highly fluctuating loop, containing the attacking cysteine residues, in the stretched unfolded state of the protein. Our results point towards proximity as a major constraint of thiol-disulfide isomerization within this immunoglobulin and potentially within other proteins.

## Results

### Forced unfolding of I27 yields isomerization-prone conformations

**Figure 2:**
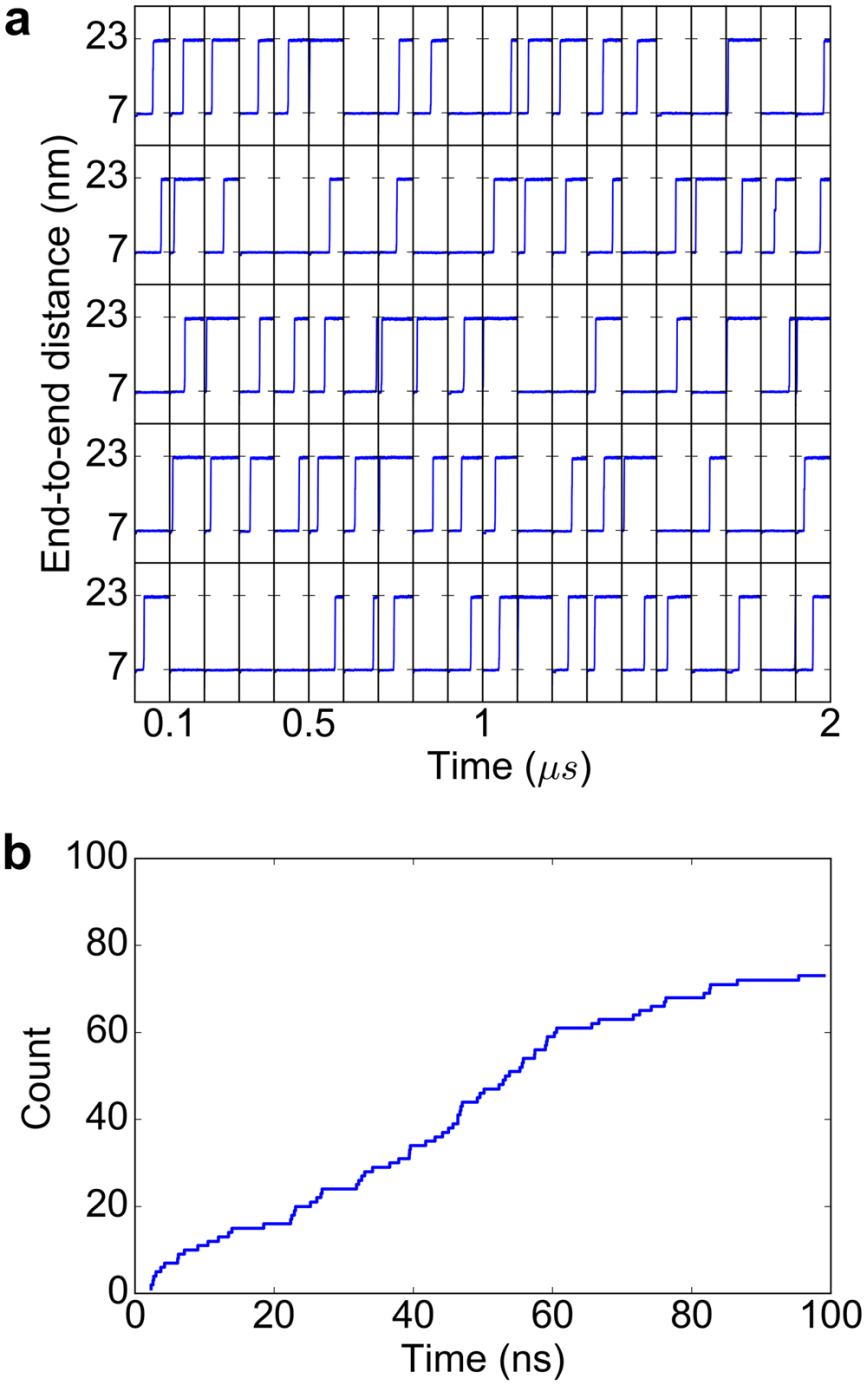
I27* unfolds at 480pN in the majority of force-clamp MD simulations. (**a**) End-to-end distances as a function of time for all 100 simulations. Each horizontal panel comprises 20 simulations, 100 ns each, separated by vertical lines, resulting in 10 µs of cumulative simulation time. (**b**) Cumulative frequency graph representing the time of unfolding for 73 out of the 100 force-clamp MD simulations for which unfolding was observed.

The wild-type I27 domain consists of 89 amino acids including two cysteines, 47Cys and 63Cys, both in the reduced state (PDB id. 1WAA, ref. 29). To monitor thiol-disulfide exchange within the protein, we introduced three additional cysteines 24Cys, 32Cys and 55Cys (Fig. 1b), resulting in a system which allowed the same thiol-disulfide reactions as in the force-clamp experiments^19^. This resulted in one disulfide bond, between 24Cys–55Cys, and three free cysteines 32Cys, 47Cys, and 63Cys. In the following, we call this mutant I27*. In 100 force-clamp MD simulations, we subjected I27* to 480 pN of force acting on the protein termini, a force just high enough to monitor unfolding on our MD time scale of tens of nanoseconds. Unfolding was reflected by a steep increase of 16 nm in end-to-end distance from 7 nm, in the folded state, to 23 nm, in the unfolded but natively disulfide bonded state (Fig. 2a). This increment is consistent with the extension of about 15 nm observed in the AFM experiments, resulting from an elongation of 11 nm in the initial force ramp plus a subsequent 4 nm stretching upon reduction of the disulfide bond 32Cys–75Cys, the first event observed in 95% of the measurements (see Fig. S2 in Cebollada *et al*.^19^). We observed unfolding events in 73 out of 100 (73%) unfolding events within 10 µs of cumulative simulation time (Fig. 2b).

**Figure 3:**
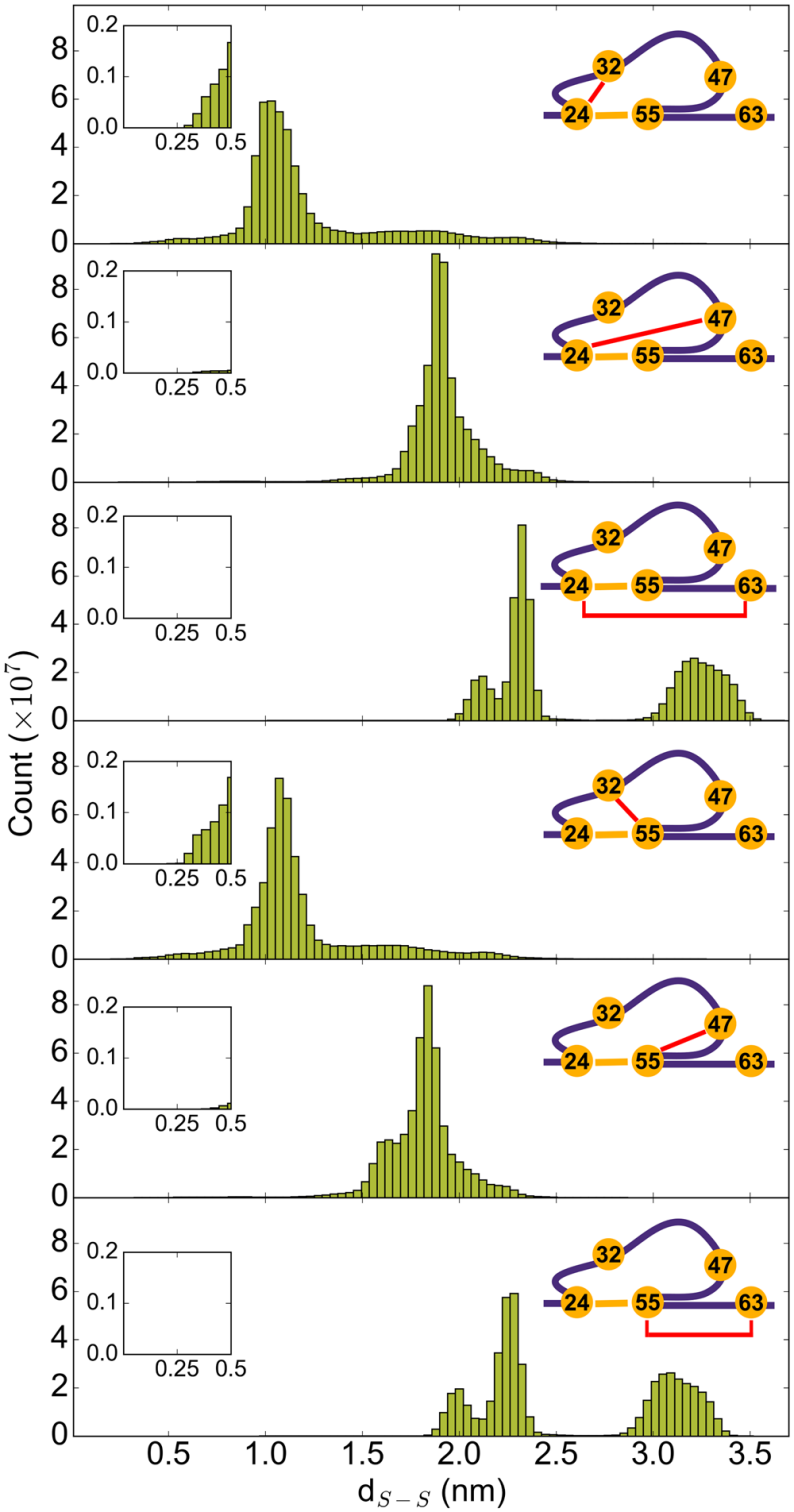
Accessibility of the disulfide bond by the free thiols in I27* strongly varies. Distributions of distances, d_S-S_, between the sulfur of one of the free cysteines (32Cys, 47Cys or 67Cys) and the sulfur of one of the disulfide bonded cysteines (24Cys or 55Cys). To guide the eye, the distance monitored is highlighted with the red line on the right scheme of each panel. Inset: close-up for distances lower than 0.5 nm.

The three-dimensional arrangement of the β-strands in the folded I27* locks the cysteines in their respective position, rendering thiol-disulfide exchange reaction impossible. The average sulfur-sulfur distance (d_S-S_) between the reduced cysteines 32Cys, 47Cys and 63Cys and the disulfide bond was approximately 1.2 nm, 2.2 nm and 2.1 nm, respectively (Figs 1b and 3). Upon I27* unfolding, the β-sheets almost completely disintegrated, and only the loop within the disulfide bond (24Cys–55Cys) remained protected from complete stretching (Fig. 1b).

Because 63Cys is located outside the loop, it was instantaneously pulled away from the disulfide bond. This is reflected in a bimodal distribution for the distance between 63Cys and the disulfide-bonded cysteines, with small distances sampled while I27* was folded, and with large distances visited after the protein was stretched (Fig. 3). Therefore, obviously, 63Cys is not a candidate for attacking the disulfide bond. We tested if the two other cysteines, 32Cys and 47Cys, which are positioned inside the protected loop, could potentially get closer to the disulfide bond and attack it (Fig. 1b). The 47Cys sampled large d_S-S_, mostly between 1.5 nm and 2.5 nm (Fig. 3). It rarely approached the disulfide bond close enough for the reaction to occur (Fig. 3, inset). On the other hand, 32Cys features distances to the bridged cysteines 24Cys and 55Cys as low as 0.25 nm (see long tails towards very short distances in the 32Cys–24Cys and 32Cys–55Cys distance distributions in Fig. 3, inset and Movie S1). A distance d_S-S_ of 0.25 nm is very close to the transition state distance of ~0.24 nm^20, 22^. Therefore, within the time scale of our simulations, of microseconds, the only reduced cysteine which got close enough to be able to attack the disulfide bond was 32Cys. This is in direct agreement with experiments, which observed thiol-disulfide exchange exclusively with 32Cys as the nucleophile^19^.

### Conformational entropy of the highly dynamic loop defines the attacking cysteine

**Figure 4:**
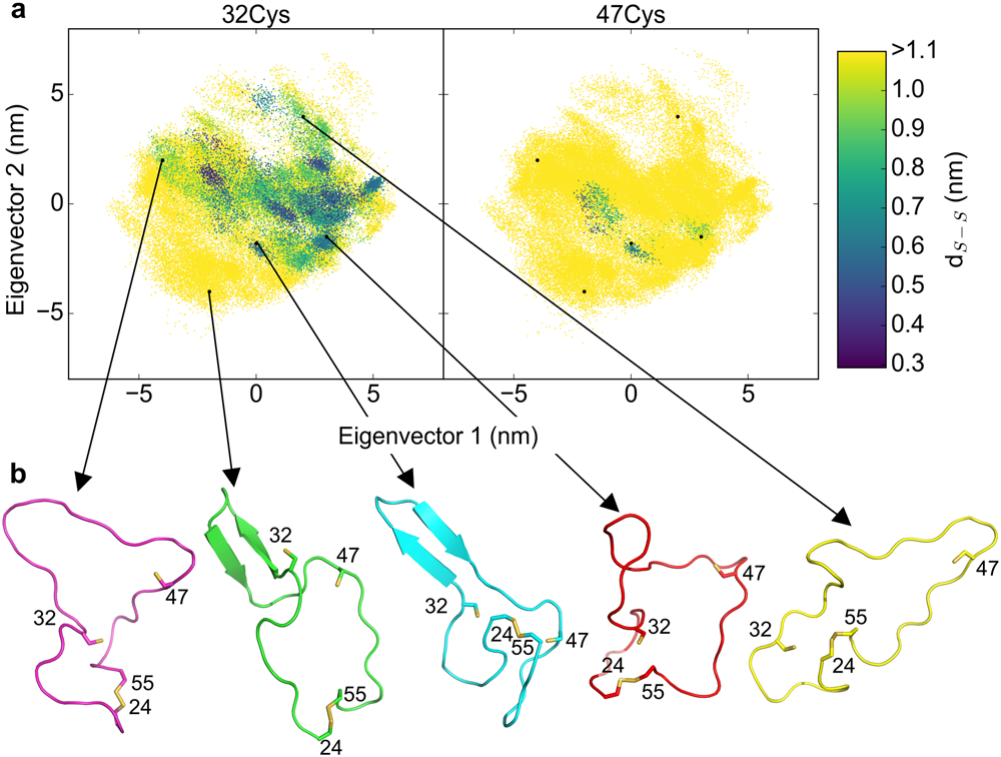
Attack of the disulfide bond by 32Cys does not compromise the wide conformational space available to the loop. (**a**) Projection of loop dynamics along the first two eigenvectors obtained from principle component analysis (PCA) colored according to the distance between 32Cys (left) or 47Cys (right) and the center-of-mass of the disulfide bond. (**b**) Typical conformations representing the width of conformational space along the two major eigenvectors.

To determine the factors underlying the pronounced preference of 32Cys over 47Cys to approach the disulfide bond, we projected the dynamics of the loop onto the two major collective modes of motion, as obtained from principal component analysis (PCA) (Fig. 4). As expected, the loop explored a broad range of conformations, reflecting its highly dynamic disordered nature (Fig. 4a). This posed the question of how these large fluctuations can prime only one of the cysteines for attack. We addressed this question by mapping the d_S-S_ distance onto the PCA projection. 32Cys is in close proximity to the disulfide bond for many different conformations of the loop, while 47Cys is close to the disulfide in only a few isolated cases (compare the size of the 2D-projection for distances smaller than 0.6 nm for 32Cys with that of 47Cys in Fig. 4a). The conformational entropy is thus higher for the state in which 32Cys approaches the disulfide bond. Thus, the approach of 32Cys appears to be more likely because the conformational fluctuations of the dynamic loop are much less constrained by small 32Cys-disulfide distances.

### Simulations reproduce regioselectivity of thiol-disulfide exchange

**Figure 5:**
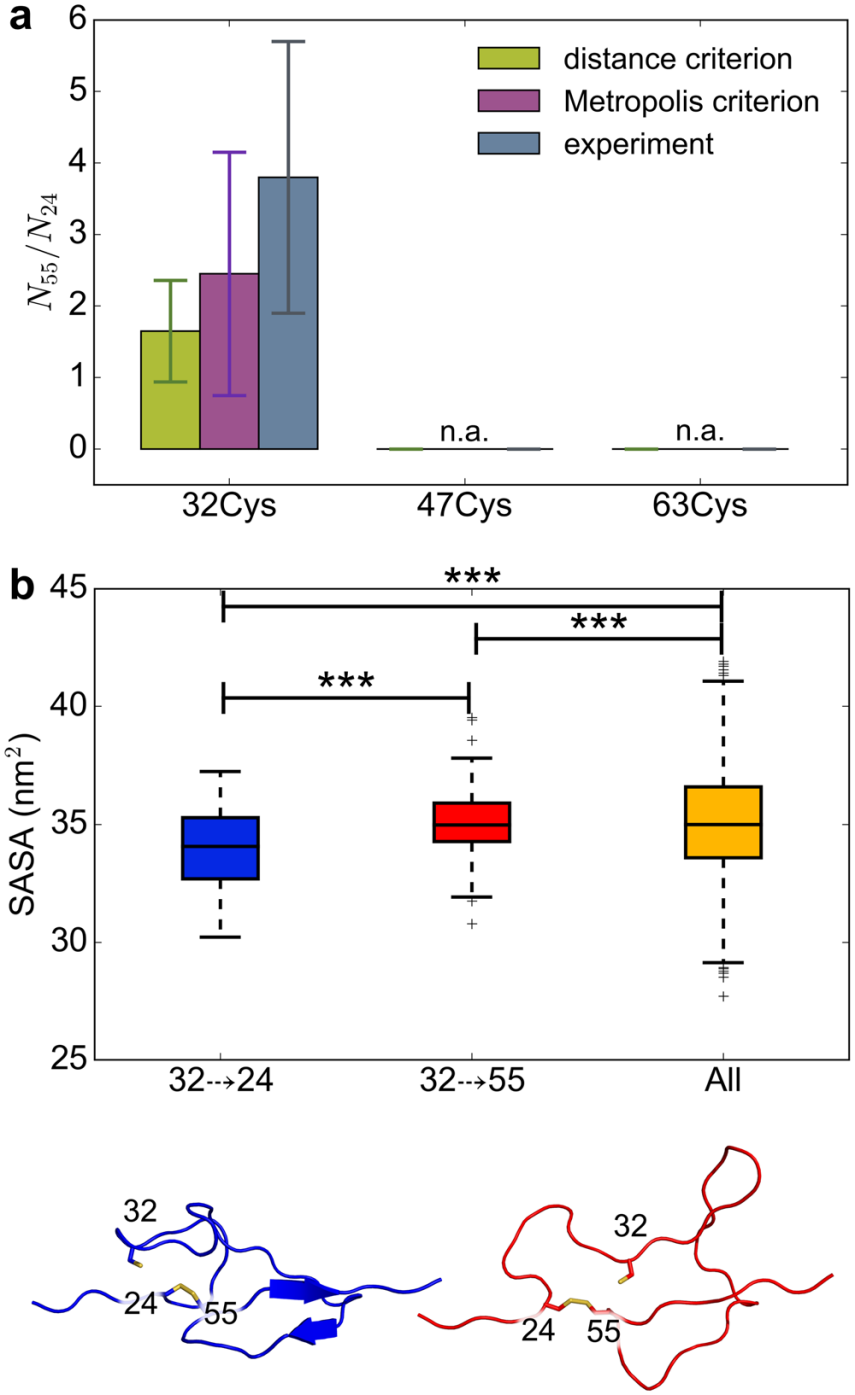
Thiol-disulfide exchange in I27* is regioselective for steric reasons. (**a**) Ratio of 55Cys (N_55_) and 24Cys (N_24_) swap counts, calculated with the distance criterion (green) and with the energy-based Metropolis criterion (magenta). The standard error was obtained by bootstrapping. The experimental ratios (gray) were taken from Alegre-Cebollada *et al*.^19^. Data for a distance cut-off of 0.5 nm is shown (see SI, Figs S3 and S4 for other distances). Counts of swaps based on distance criterion were obtained from 100 simulations, while those based on the Metropolis criterion from a subset of 20 simulations. (**b**) Solvent accessible surface area (SASA) of conformations allowing a 32–>24 and a 32–>55 S_N_2 reaction, and of all conformations. *** denotes p < 0.001. Bottom: a representative conformation for a 32–>24 (blue) and 32–>55 (red) reaction.

We next asked if the simulations could predict the regioselectivity observed experimentally, i.e. a preference of 32Cys for the nucleophilic attack of one over the other cysteines in the disulfide bond. To this end, as a first simple approximation, we defined any approach of one of the free thiols to one of the disulfide sulfur atoms below 0.5 nm as a successful S_N_2 reaction, resulting in the formation of a new disulfide bond. According to this definition, 32Cys reacted with 55Cys in 18 simulations and with 24Cys in 12 simulations resulting in a 1.5-fold regioselectivity of 55Cys versus 24Cys (Fig. 5a). Encouragingly, we observed a pronounced preference of 32Cys reacting with 55Cys independently of the distance cut-off (Supplementary Figure S3). The only exception was found for very small cut-offs (0.3 nm and smaller), which is probably a consequence of the small sample size (less than 6 events) at these cut-offs. In order to assess the feasibility of the S_N_2 reaction to occur, in addition to a mere distance criterion, we also used an energy-based Metropolis criterion, such that a swap was only accepted if the potential energy of the energy-minimized configurations before and after swapping compared favorably with one another (see Methods for details). Using this criterion, we again recovered the trend in regioselectivity, with 55Cys being preferred over 24Cys, 2.5-fold at a cut-off of 0.5 nm (Fig. 5a). Thus, introducing an energetic criterion for the occurrence of thiol-disulfide exchange reaction does not change the predicted regioselectivity, again pointing towards accessibility as the major determinant of thiol-disulfide exchange in I27*. The reaction between 32Cys and 55Cys observed by AFM occurred approximately 3.8 times more often than the same reaction between 32Cys and 24Cys, which is in close agreement with our prediction (Fig. 5a). Due to the relatively small number of exchange reactions observed in both experiments and simulations, we cannot identify the most predictive distance cut-off or energy criterion to decide on isomerization. However, additionally introducing the Metropolis criterion rendered the prediction of the regioselectivity more robust regarding the distance criterion (Supplementary Figure S4).

S_N_2 reactions are known to proceed along a ~180 degree S-S-S angle (even though our findings hint towards angles that can also be lower than 180 degree^22^. We analyzed the angle distributions of the three sulfurs involved in the reaction once the distance of the disulfide attacking sulfur becomes small (<0.5 nm). Interestingly, they cover a sub-range from 80–180 degree (Supplementary Figure S5a). If we only consider S_N_2 reactions to occur for angles larger than a given lower boundary value θ, we do not see a substantial difference in the probability P(angle > θ) of Cys55 compared to that of Cys24, apart from a slightly larger value for Cys55 over Cys24 for θ between 90 and 130 degree (Supplementary Figure S5b). The probability for obtaining large angles is in consequence roughly the same (Supplementary Figure S5c). Therefore, considering the attack angle as a criterion does not seem to substantially modify the preference, already determined by the distance, of 55 over 24 for the swap. Note that only few conformations displayed angles larger than 135 degree. Therefore, for such large angles, we attribute the large variations in the probability ratio to a large statistical error rather than to a physico-chemical feature of the S_N_2 reaction.

### Conformational space of the loop determines reaction regioselectivity

Having validated the predictive power of our simulation protocol with regard to the preferred reacting sulfur atoms, we analyzed the molecular determinants of the observed regioselectivity. To this end, we compared reactive conformations, i.e. those with 32Cys in the proximity of 24Cys (denoted 32–>24) or 55Cys (32–>55) to all conformations (Fig. 5b). We observed that the solvent accessible surface area (SASA) of 32–>24 conformations was significantly smaller than the SASA of 32–>55 structures (Fig. 5b). Thus, for 32Cys to approach the 24Cys the remaining loop had to be more compact (Fig. 5b, left red structure), while the loop remains in more expanded conformations for 32Cys attacking 55Cys, similar to the average expansion of the loop (Fig. 5b, right blue structure). Taken together, conformations 32–>55, which have a high propensity to form a new 32–55 disulfide bond, highly resemble the vast majority of conformations. In sharp contrast, thiol-disulfide exchange of 32Cys approaching 24Cys requires a rarely sampled collapsed conformation.

## Discussion

Thiol-disulfide exchange is an extremely important and specific reaction *in vivo* ^30, 31^. Our current mechanistic understanding of thiol-disulfide exchange in proteins is largely restricted to enzymatic reactions. Enzymes for thiol-disulfide exchange have been tuned during evolution towards specific redox potentials, reaction kinetics and mechanisms of substrate recognition^32, 33^. Instead, intramolecular thiol-disulfide exchange might be very different in nature, as a specialized active site is lacking. It is currently unknown if the thiol nucleophile is specifically activated for the S_N_2 reaction by a lowered pK_a_, if certain base-catalysis must be at play, or if the mere proximity already allows the reaction to take place. In this study, we performed multiple microseconds of force-clamp MD simulations on the mutant I27* to understand the underlying rationale for the selective thiol-disulfide exchange reactions induced by mechanical force. Previous AFM experiments^19^ demonstrated this to be a highly suitable model for studying thiol-disulfide exchange because the intramolecular and irreversible reaction can be followed on a single-molecule level.

Our simulations allowed to monitor the dynamics of the free cysteines (32Cys, 47Cys, and 63Cys) in I27* under force and thereby to give insight into the molecular mechanism underlying force-induced thiol-disulfide exchange with the disulfide bond 24Cys–55Cys. As a first major prerequisite to observe exchange, unfolding of I27* had to take place. This releases the structural constraints of the free cysteines imposed by the secondary structure of the protein and thereby allow them to freely move and potentially attack the disulfide bond. Unfolding forces pulled away 63Cys and therefore impeded this residue to act as a reactive nucleophile. Instead, 32Cys and 47Cys, both contained in the loop enclosed by the disulfide upon unfolding, were in this respect equally likely to play the role of the attacking cysteine within the disordered loop. We were able to correctly predict 32Cys as the cysteine preferentially attacking the disulfide bond. As the underlying molecular mechanism, our data support the larger conformational entropy of a reactive configuration (obtained at short sulfur-sulfur distances) given by the intrinsic dynamics of the loop to be the main factor defining 32Cys as the attacking cysteine. Enthalpy can not be ruled out to also influence which cysteines attack which sulfur in the disulfide, but in the simulations it was found to be a highly fluctuating quantity, from which we could not obtain significant differences. This is in contrast to the structural (interpreted as entropic) significant size between the 32Cys-type preferred conformational ensemble over that for 47Cys-type.

The agreement in the elongation upon unfolding observed in our simulations (~16 nm) with that measured in the AFM experiments (~15 nm^19^) supports the notion that despite of its high magnitude, the applied external force in our simulations did not alter the resulting isomerization-prone topological conformation. In addition, while the stretched termini remained under tension, we expect the loop to be tension free and it is in this low-tension state in which we think the cysteines attacked the disulfide bond. An argument supporting the low-tension for the loop is its high flexibility captured in the simulations despite of the applied external load. Furthermore, there is good agreement between our simulations and the previous AFM experiments^19^ on which cysteine gets more often closer to the disulfide bond. In consequence, If the tension on the termini modified the conformational dynamics of the loop, we consider its effect on the accessibility only to be small. The use of a high force in fact accelerated the kinetics of the unfolding process, but the rates and unfolding pathways can be reconciled with those obtained at low (or zero) force as determined in our recent publication for another protein^34^. For all these reasons we think that although the pulling protocol (in particular the magnitude of the applied force) drastically modulate the kinetics of the process, it may not substantially change our findings about the resultant isomerization-prone conformations and their populations.

Our results also reproduced the regioselectivity of the reaction, with 32Cys preferring 55Cys compared to 24Cys. The regioselective reaction was observed using only a simple distance criterion (i.e. distance between the attacking 32Cys and cysteines forming the disulfide bond). The observed ratio of 1.7 between 32Cys approaching 55Cys and 32Cys approaching 24 Cys (N_55_/N_24_, Fig. 5a) is in accordance with experimental results. For a typical S_N_2 reaction, the better leaving group is 55Cys because of a nearby positively charged 54Lys, which should lower the thiol group pK_a_ value^19^. Thus, intuitively the preferred reaction should be between 32Cys and 24Cys, which is not the case. Instead, a simple steric contribution is sufficient to explain the experimental observation. Namely, for most of the conformational space explored by the flexible loop, 55Cys is easily accessible, while the attack of 24Cys is only feasible for a subset of rarely observed collapsed loop conformations.

We also mimicked the reaction by changing the bonds between sulfur atoms based on a Metropolis energy criterion (Movie S1). This approach accounted, in first approximation, for the energetic cost to achieve the equilibrated conformational state after the reaction takes place. However, adding this information did not substantially modify the regioselectivity observed by using a simple distance criterion (changes within the error in Fig. 5a). This corroborates the notion of the relative accessibility of the reactants -prior to the reaction- as the primary determinant of thiol-disulfide isomerisation in the mutant I27*. Because the Metropolis criterion was based on the potential energy, this result also suggest that enthalpy plays a small role on the regioselectivity. Local environment changes in the reaction center and subsequent energetic details during the reaction thus seem to play a minor fine-tuning role in this protein. A polarizable force field or combined quantum mechanical/molecular mechanical approach would be needed to address the question in further detail how nearby protein charges might further alter the observed ratios.

We here deliberately did not incorporate information on the free energy barrier or transition state structure of the chemical reaction, and yet our method exhibited predictive power. This is similar to recent results on oxidative protein folding using a coarse-grained self-organized polymer model that has been extended to model redox reactions^10^. Note that little work has been done on the development of non-covalent sulfur-sulfur molecular mechanics (MM) force-field parameters. Actually, conventional non-bonded parameters used here for sulfur atoms come back to the first versions of the Amber force-field (Amber94^35^). This may be attributed to the fact that typical protein interactions include many of carbon-sulfur or water-sulfur but rarely sulfur-sulfur interactio﻿ns. Sulfur-sulfur interactions should be assessed more carefully by comparison to quantum mechanical (QM) calculations. However, this comparison goes beyond the scope of our manuscript. Nevertheless, given that the Lennard-Jones and Coulomb interactions are pairwise, the S-H-S-S contacts for both 31Cys and 47Cys are expected to contribute with the same amount to the potential energy and thereby to introduce the same force-field error. This suggests that the reactant selection observed here is independent of the specific sulfur-sulfur parameters. We also want to stress that the MC step was only intended to confirm roughly that the reaction occurs in line with the thermodynamic Boltzmann weights observed in the experiments. Our approach is limited in the sense that it does not provide information about the kinetic of the process. A more complete picture of non-enzymatic thiol-disulfide isomerization in proteins could only be obtained by coupling the large-scale conformational changes to hybrid quantum/molecular mechanical (QM/MM) simulations, e.g. by a switching between an MM and QM/MM description based on a distance criterion. This approach could potentially account for both the kinetics and thermodynamics of the exchange process. Nevertheless, for the particular case of I27*, our observations and previous experiments predict that the regioselectivity is conditioned by the attempt frequency (which is dependent on the protein conformational dynamics) rather than by the free energy barriers for the different putative reaction partners.

## Conclusion

In conclusion, our simulation data correctly predicted the regioselectivity of the thiol-disulfide isomerisation in the immunoglobulin I27* mutant. Intriguingly, for this specific case, the most important determinant for the thiol-disulfide reaction to occur was accessibility of the reactant, which was given by the intrinsic motions of the protein. This is consistent with the simple “proximity rule” proposed by Camacho and Thirumalai^36, 37^ which is based on the assumption that disulfide bond formation is a random process depending only on the peptide length. Furthermore, our simulations (which fully include non-covalent interactions in an explicit water model) suggest that such details do not seem to play a significant role deciding on the reacting species for IG27*, further confirming the generality of the proximity rule. Thiol-disulfide exchange upon forced unfolding has been recently observed for a growing number of proteins such as von Willebrand factor^17, 18, 38^. Whether they follow the same mechanism proposed here for I27* remains to be elucidated.

## Methods

### Equilibrium MD simulation setup

Simulations were performed using GROMACS version 5.0^39^. The Amber99sb-ildn*^40, 41^ force field was used for the protein together with the modified ion parameters by Joung and Cheatham^42^ and the TIP3P water model^43^. To avoid infinitely large forces at smaller non-bonded sulfur-sulfur (d_S-S_) distances, potentially visited during simulations, we used a softcore potential for sulfur atoms with alpha = 0.3, lambda = 0.9 and power = 1. Note that the softcore and normal Lennard-Jones interactions are identical at distances beyond 0.35 nm, nearby the potential well minima, where most sampled distances in the simulation were found. The crystal structure of the I27 protein domain (pdb: 1WAA^29^) was mutated with PyMol^44^ to introduce three additional cysteines: 24Cys, 32Cys and 55Cys. The 24Cys and 55Cys cysteines were oxidized to form a disulfide bond, while 32Cys remained as a free thiol. The protein was centered in a dodecahedron box (size 8.7 × 8.7 × 6.2 nm^3^) and solvated by approximately 15,000 water molecules. Ions (Na^+^ and Cl^−^) were added to achieve a final concentration of 0.15 M, with an excess of Na^+^ to obtain an electroneutral system. The system was energy minimized using the steepest descent algorithm. Next, the solvent was equilibrated during 4 ns, with the protein heavy atoms restrained by harmonic springs with a force constant of 1000 kJmol^−1^ nm^−2^. A constant temperature of 300 K and a constant pressure of 1 bar were ensured by employing the Berendsen^45^ thermostat (coupling constant τ = 0.1 ps) and barostat (coupling constant τ = 1.0 ps). During the production run, the protein restraints were released and the Berendsen thermostat and barostat were replaced by the v-rescale thermostat^46^ with τ of 0.5 ps and the Parrinello-Rahman barostat^47^ with τ of 5.0 ps, respectively. The group-based cut-off scheme was used for treating short-range non-bonded interactions. A user specified tabulated Lennard-Jones potential was employed for calculating short-range van der Waals interactions with a distance cut-off of 1 nm. Long-range electrostatic interactions were computed by the particle mesh Ewald method^48^. An integration time step of 2 fs was achieved by adding LINCS constraints^49^ to the bonds involving hydrogen atoms in the protein and Settle^50^ to all bonds and angles of the water molecules. The production run was 200 ns long.

### Force-clamp MD simulation setup

Twenty representative Ig27 conformations were extracted from the last 150 ns of the equilibrium MD simulation. A clustering analysis was carried out to select these conformations. Conformations were assigned to belong to one cluster if the root-mean-square deviation of their backbone atoms was smaller than 0.1 nm. Subsequently, one conformation was selected as a representative from each cluster. For each starting conformation, the protein was centered in a box of size 30.0 × 7.0 × 7.0 nm^3^ and it was solvated with approximately 48,000 water molecules. Na^+^ and Cl^−^ ions were also added to achieve an electroneutral system at a NaCl concentration of 0.15 M. The α-carbon atom of one termini was kept fixed at its initial position and the center-of-mass of the other terminal amino acid was pulled away with a constant force of 480 pN. The magnitude of the pulling force was chosen such that the unfolding of I27* domain occurred in a few tens of nanoseconds (Supplementary Figure S1). The same force field parameters and simulation settings were applied as in the equilibrium MD simulation. Altogether we performed 100 × 100 ns of force-clamp MD simulations, yielding 10 µs of concatenated simulation time. Trajectories were written with an output frequency of 20 fs to ensure high precision in the highly fluctuating cysteine positions.

### Criteria for thiol-disulfide swapping

To find conformations in which disulfide bonds could potentially swap we used two methods: (1) distance-based or (2) distance- and energy-based. When using (1) the distance-based criterion, the distance between each of the S atoms of free thiols and the S atoms of cysteines in the disulfide bond (d_S-S_) was measured at each time step. The definition of a successful swap was the first time that the d_S-S_ was lower than a certain threshold. Only the first instance of a small distance is relevant because the stretching force renders the reaction irreversible. When using (2), the distance criterion was coupled with the energy-based Metropolis criterion^50^, then each time that d_S-S_ was lower than a threshold value, the Metropolis criteria was applied (Supplementary Figure S2). The product was obtained by swapping the disulfide bond i.e. assigning the new disulfide bond, removing the previous one and moving the thiol hydrogen to the leaving group of the product. Next, the conformation was relaxed by performing local minimization with the steepest descent algorithm. Only amino acid and waters in a radius of 0.5 nm around the three reactive cysteines were allowed to move, while all other atoms of the system were frozen. The reactant structure was also energy minimized. Finally, the potential energies after minimization were compared and a swap was decided based on a Metropolis criterion. The procedure was repeated in subsequent MD steps until the Metropolis criterion was accepted. Because the Metropolis criterion only served as an additional confirmation and required the saving of large trajectories including all particles and frames, we chose a subset of 20 trajectories for this analysis.

Principal component analysis – PCA^52^ - was performed to detect essential collective motions of the loop, which remained after unfolding of I27*. Only the trajectories that exhibited unfolding were considered. The first part of these trajectories, where the protein was still folded (i.e. when the N- to C-terminus distance was smaller than 20 nm) was discarded. Only backbone atoms of the loop (residues 24 to 55) were considered. Positions were monitored at a frequency of 20 ps. Optimal superposition of the conformations was carried out before PCA to minimize the variance over the ensemble of conformations and thereby remove ambiguous rotations rising in very flexible molecules^53^. The first two eigenvectors obtained for the loop dynamics accounted for almost 50% of the total positional fluctuations. Both PCA and the optimal superposition were carried out using GROMACS tools.

Solvent accessible surface area (SASA) was calculated using GROMACS SASA utility^54^. The SASA was computed every 2 ns, a time period for which the SASA autocorrelation was zero. To determine the significance of differences between distributions a Kolmogorov–Smirnov test as implemented in SciPy^55^ was applied. Only independent events were used for this test.

## Electronic supplementary material

Supplementary Information

Movie S1

## References

[1] Thornton JM (1981). Disulphide bridges in globular proteins. J. Mol. Biol..

[2] Betz SF (1993). Disulfide bonds and the stability of globular proteins. Protein Sci. Publ. Protein Soc..

[3] Cook KM, Hogg PJ (2013). Post-translational control of protein function by disulfide bond cleavage. Antioxid. Redox Signal..

[4] Kosuri P (2012). Protein folding drives disulfide formation. Cell.

[5] Oka OBV, Bulleid NJ (2013). Forming disulfides in the endoplasmic reticulum. Biochim. Biophys. Acta.

[6] Hogg PJ (2003). Disulfide bonds as switches for protein function. Trends Biochem. Sci..

[7] Welker E, Wedemeyer WJ, Narayan M, Scheraga HA (2001). Coupling of conformational folding and disulfide-bond reactions in oxidative folding of proteins. Biochemistry (Mosc.).

[8] Bronsoms S (2011). Oxidative folding and structural analyses of a Kunitz-related inhibitor and its disulfide intermediates: functional implications. J. Mol. Biol..

[9] Lewney S, Smith LJ (2012). Characterization of an alternative low energy fold for bovine α-lactalbumin formed by disulfide bond shuffling. Proteins.

[10] Qin M, Wang W, Thirumalai D (2015). Protein folding guides disulfide bond formation. Proc. Natl. Acad. Sci. USA.

[11] Arai K, Kumakura F, Iwaoka M (2010). Characterization of kinetic and thermodynamic phases in the prefolding process of bovine pancreatic ribonuclease A coupled with fast SS formation and SS reshuffling. Biochemistry (Mosc.).

[12] Patel S, Chaffotte AF, Amana B, Goubard F, Pauthe E (2006). *In vitro* denaturation-renaturation of fibronectin. Formation of multimers disulfide-linked and shuffling of intramolecular disulfide bonds. Int. J. Biochem. Cell Biol..

[13] Ahamed J (2008). *In vitro* and *in vivo* evidence for shear-induced activation of latent transforming growth factor-β1. Blood.

[14] Ganderton T, Berndt MC, Chesterman CN, Hogg PJ (2007). Hypothesis for control of von Willebrand factor multimer size by intra-molecular thiol-disulphide exchange. J. Thromb. Haemost. JTH.

[15] Feughelman, M. *Mechanical Properties and Structure of Alpha-keratin Fibres: Wool, Human Hair and Related Fibres* (UNSW Press, 1997).

[16] Weigmann H-D, Rebenfield L, Dansizer C (1966). Kinetics and Temperature Dependence of the Chemical Stress Relaxation of Wool Fibers. Text. Res. J..

[17] Shapiro SE (2014). The von Willebrand factor predicted unpaired cysteines are essential for secretion. J. Thromb. Haemost. JTH.

[18] Solecka BA, Weise C, Fuchs B, Kannicht C (2016). Free thiol groups in von Willebrand factor (VWF) are required for its full function under physiological flow conditions. Thromb. Res..

[19] Alegre-Cebollada J, Kosuri P, Rivas-Pardo JA, Fernández JM (2011). Direct observation of disulfide isomerization in a single protein. Nat. Chem..

[20] Fernandes PA, Ramos MJ (2004). Theoretical insights into the mechanism for thiol/disulfide exchange. Chem. Weinh. Bergstr. Ger..

[21] Neves RPP, Fernandes PA, Varandas AJC, Ramos MJ (2014). Benchmarking of Density Functionals for the Accurate Description of Thiol-Disulfide Exchange. J. Chem. Theory Comput..

[22] Li W, Gräter F (2010). Atomistic evidence of how force dynamically regulates thiol/disulfide exchange. J. Am. Chem. Soc..

[23] Bach RD, Dmitrenko O, Thorpe C (2008). Mechanism of thiolate-disulfide interchange reactions in biochemistry. J. Org. Chem..

[24] Carvalho ATP (2008). Mechanism of thioredoxin-catalyzed disulfide reduction. Activation of the buried thiol and role of the variable active-site residues. J. Phys. Chem. B.

[25] Levin L (2013). A single disulfide bond disruption in the β3 integrin subunit promotes thiol/disulfide exchange, a molecular dynamics study. PloS One.

[26] Quan S, Schneider I, Pan J, Von Hacht A, Bardwell JCA (2007). The CXXC motif is more than a redox rheostat. J. Biol. Chem..

[27] Li W, Baldus IB, Gräter F (2015). Redox potentials of protein disulfide bonds from free-energy calculations. J. Phys. Chem. B.

[28] van Duin ACT, Dasgupta S, Lorant F, Goddard WA (2001). ReaxFF:  A Reactive Force Field for Hydrocarbons. J. Phys. Chem. A.

[29] Stacklies W, Vega MC, Wilmanns M, Gräter F (2009). Mechanical network in titin immunoglobulin from force distribution analysis. PLoS Comput. Biol..

[30] Gilbert HF (1990). Molecular and cellular aspects of thiol-disulfide exchange. Adv. Enzymol. Relat. Areas Mol. Biol..

[31] Gilbert HF (1995). Thiol/disulfide exchange equilibria and disulfide bond stability. Methods Enzymol..

[32] Collet J-F, Messens J (2010). Structure, Function, and Mechanism of Thioredoxin Proteins. Antioxid. Redox Signal..

[33] Marino SM, Gladyshev VN (2012). Analysis and Functional Prediction of Reactive Cysteine Residues. J. Biol. Chem..

[34] Costescu BI, Sturm S, Gräter F (2017). Dynamic disorder can explain non-exponential kinetics of fast protein mechanical unfolding. J. Struct. Biol..

[35] Cornell WD (1995). A Second Generation Force Field for the Simulation of Proteins, Nucleic Acids, and Organic Molecules. J. Am. Chem. Soc..

[36] Camacho CJ, Thirumalai D (1995). Theoretical predictions of folding pathways by using the proximity rule, with applications to bovine pancreatic trypsin inhibitor. Proc. Natl. Acad. Sci. U. S. A..

[37] Camacho CJ, Thirumalai D (1995). Modeling the role of disulfide bonds in protein folding: entropic barriers and pathways. Proteins.

[38] Ganderton T, Wong JWH, Schroeder C, Hogg PJ (2011). Lateral self-association of VWF involves the Cys2431-Cys2453 disulfide/dithiol in the C2 domain. Blood.

[39] V D Spoel D (2005). GROMACS: fast, flexible, and free. J. Comput. Chem..

[40] Best RB, Hummer G (2009). Optimized molecular dynamics force fields applied to the helix-coil transition of polypeptides. J. Phys. Chem. B.

[41] Lindorff-Larsen K (2010). Improved side-chain torsion potentials for the Amber ff99SB protein force field. Proteins.

[42] Joung IS, Cheatham TE (2008). Determination of alkali and halide monovalent ion parameters for use in explicitly solvated biomolecular simulations. J. Phys. Chem. B.

[43] Mark P, Nilsson L (2001). Structure and Dynamics of the TIP3P, SPC, and SPC/E Water Models at 298 K. J. Phys. Chem. A.

[44] *The PyMOL Molecular Graphics System*. (Schrödinger, LCC, 2010).

[45] Berendsen HJC, Postma JPM, Gunsteren WF, van DiNola A, Haak JR (1984). Molecular dynamics with coupling to an external bath. J. Chem. Phys..

[46] Bussi G, Donadio D, Parrinello M (2007). Canonical sampling through velocity rescaling. J. Chem. Phys..

[47] Parrinello M, Rahman A (1981). Polymorphic transitions in single crystals: A new molecular dynamics method. J. Appl. Phys..

[48] Abraham MJ, Gready JE (2011). Optimization of parameters for molecular dynamics simulation using smooth particle-mesh Ewald in GROMACS 4.5. J. Comput. Chem..

[49] Hess B, Bekker H, Berendsen HJC, Fraaije JGEM (1997). LINCS: A linear constraint solver for molecular simulations. J. Comput. Chem..

[50] Shuichi Miyamoto & Peter A. Kollman, Settle: An analytical version of the SHAKE and RATTLE algorithm for rigid water models. *Journal of Computational Chemistry* **13**(8), 952–962 (1992).

[51] Hastings, W. K. Monte Carlo sampling methods using Markov chains and their applications. *Biometrika* **57**, 97–109 (1970).

[52] Andrea Amadei, Antonius B. M. Linssen & Herman J. C. Berendsen. Essential dynamics of proteins. *Proteins: Structure, Function, and Genetics* **17**(4), 412–425 (1993). 10.1002/prot.340170408 8108382

[53] Jones, E., Oliphant, T. & Peterson, P. *SciPy: Open Source Scientific Tools for Python* (2001).

[54] Eisenhaber F, Lijnzaad P, Argos P, Sander C, Scharf M (1995). The double cubic lattice method: Efficient approaches to numerical integration of surface area and volume and to dot surface contouring of molecular assemblies. J. Comput. Chem..

[55] Vytautas Gapsys & Bert L. de Groot. Optimal Superpositioning of Flexible Molecule Ensembles. *Biophysical Journal* **104**(1), 196–207 (2013). 10.1016/j.bpj.2012.11.003 PMC3540257 23332072

