# Supplementary material for: Accessibility explains preferred thiol-disulfide isomerization in a protein domain
Source: Sci Rep. 2017 Aug 29;7:9858. doi: 10.1038/s41598-017-07501-4 (PMC5575259; doi:10.1038/s41598-017-07501-4)
Supplement: Supplementary file 1 — Supplementary Information [file 41598_2017_7501_MOESM1_ESM.pdf]

Supporting information:

Accessibility explains preferred thiol-disulfide isomerization in a protein domain

Katra Kolšek,[†,⊥] Camilo Aponte-Santamaría,[†,‡,§,⊥] and Frauke Gräter[*,†,‡]

[†]Heidelberg Institute for Theoretical Studies, Heidelberg, Germany

[‡]Interdisciplinary Center for Scientific Computing, Heidelberg University, Heidelberg, Germany

[§]Present address: Max Planck Tandem Group in Computational Biophysics, University of Los Andes, Bogotá, Colombia

[⊥]these two authors contributed equally

*correspondence to: frauke.graeter@h-its.org

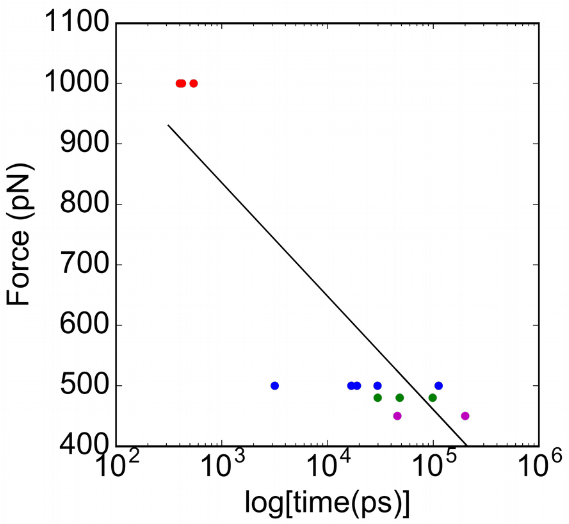

Figure S1. Force applied to protein termini in force-clamp MD simulations as a function of unfolding time. The 1000 pN – red, 500 pN blue, 480 pN – green and 450 pN -magenta with fitted black line.

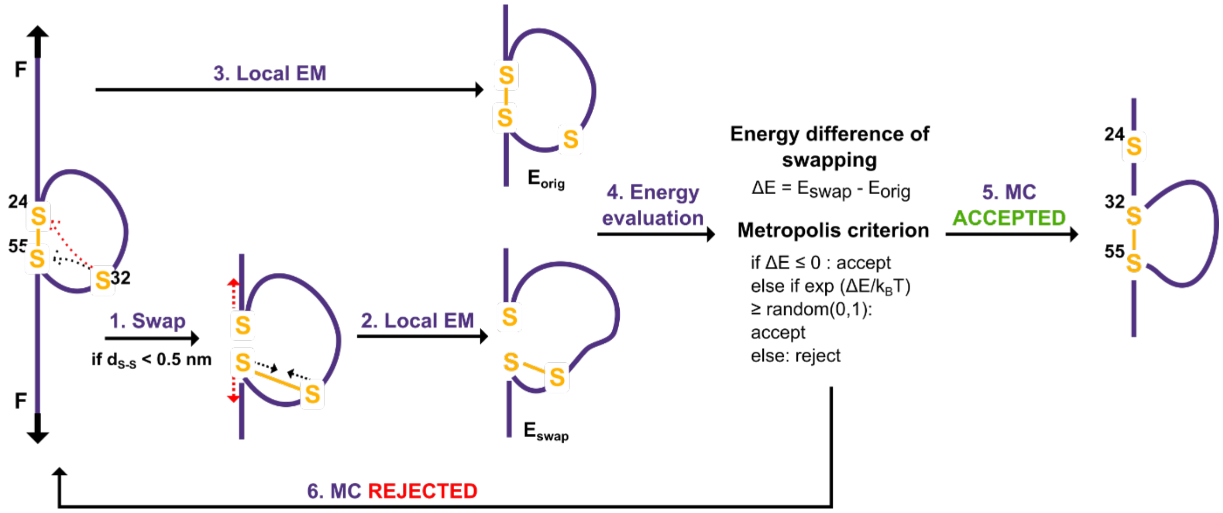

Figure S2. Schematic representation of a swapping protocol. This postprocessing method is based on a Metropolis criterion which evaluates feasibility of reaction from energy difference between reactant and product. Each snapshot with the distance (dS-S) between S atom of free cysteine (here 32Cys) and a S atom of bound cysteine (here 55Cys) is evaluated. (1) The new topology with the new disulfide bond is created. (2) The newly formed system configuration is locally minimized (see Methods) to relax bond lengths and angles. (3) At the same time the reactant structure is exposed to local energy minimization (EM), so (4) energy evaluation with Metropolis criterion (MC) can be performed on equally treated systems. If the (5) MC is accepted we assume that the thiol-disulfide exchange reaction would occur, on the other hand if (6) MC is rejected new snapshot with the dS-S lower than 0.5 nm is found and the cycle continues until the MC is accepted or the trajectory finishes.

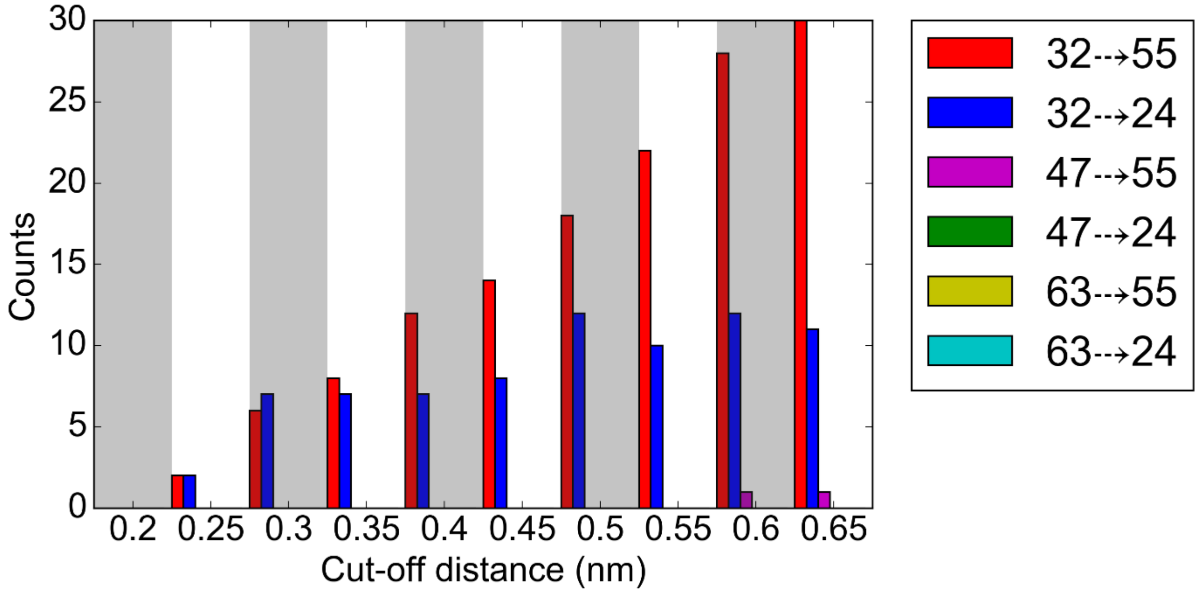

Figure S3. Number of trajectories (out of 100) with a successful swapping as given by the distance criterion, as a function of the cut-off distance.

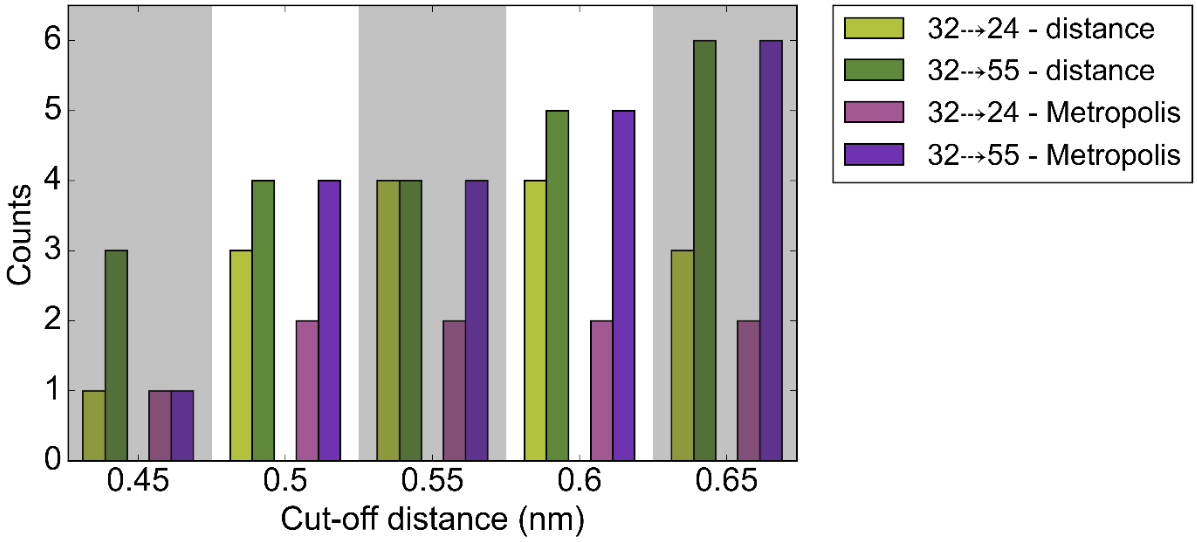

Figure S4. Number of trajectories (out of 20) with successful swapping between cysteines as denoted, depending on the distance or Metropolis criterion (see Methods), as a function of the cut-off distance.

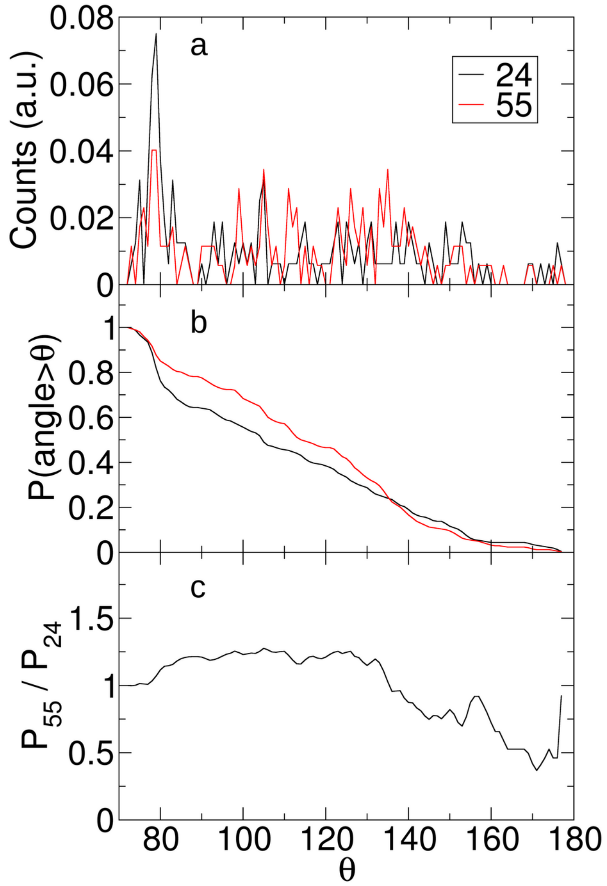

Figure S5. **A.** Distribution of angles of the three sulfur atoms involved in the reaction once the distance of the attacking disulfide (32Cys) becomes smaller than 0.5 nm. Distribution for the sulfur of 24Cys (black) and 55Cys (red) is shown. **B.** Probability that the angle between sulfurs is larger than a given lower-boundary value θ, P(angle>θ), as a function of θ. Same color format as in A. **C.** Ratio between the probabilities shown in B for 55Cys and 32 Cys.

.

Movie S1

Example of thiol-disulfide shuffling observed in the I27* protein, by our hybrid Monte Carlo and Molecular Dynamics simulation approach.
